# Choosing the best algorithm among five thyroid nodule ultrasound scores: from performance to cytology sparing—a single-center retrospective study in a large cohort

**DOI:** 10.1007/s00330-021-07703-5

**Published:** 2021-02-18

**Authors:** Clotilde Sparano, Valentina Verdiani, Cinzia Pupilli, Giuliano Perigli, Benedetta Badii, Vania Vezzosi, Edoardo Mannucci, Mario Maggi, Luisa Petrone

**Affiliations:** 1 Endocrinology Unit, Department of Experimental and Clinical Biomedical Sciences “Mario Serio”, University of Florence, Florence, Italy; 2 Endocrinology Unit, Santa Maria Nuova Hospital, Azienda USL Toscana Centro, 50122 Florence, Italy; 3 Unit of General and Endocrine Surgery, Centre of Oncological and Minimally Invasive Surgery, Department of Surgery and Translational Medicine, University of Florence, Florence, Italy; 4 Department of Histopathology and Molecular Diagnostics, Azienda Ospedaliero-Universitaria Careggi, Florence, Italy; 5 Consorzio I.N.B.B., 00136 Rome, Italy; 6 Endocrinology Unit, Medical-Geriatric Department, Azienda Ospedaliero-Universitaria Careggi, Viale Pieraccini 18, 50139 Florence, Italy

**Keywords:** Thyroid nodules, Ultrasonography, Biopsy, fine-needle, Thyroid imaging, reporting, and data system, Cytology sparing

## Abstract

**Objective:**

Incidental diagnosis of thyroid nodules, and therefore of thyroid cancer, has definitely increased in recent years, but the mortality rate for thyroid malignancies remains very low. Within this landscape of overdiagnosis, several nodule ultrasound scores (NUS) have been proposed to reduce unnecessary diagnostic procedures. Our aim was to verify the suitability of five main NUS.

**Methods:**

This single-center, retrospective, observational study analyzed a total number of 6474 valid cytologies. A full clinical and US description of the thyroid gland and nodules was performed. We retrospectively applied five available NUS: KTIRADS, ATA, AACE/ACE-AME, EUTIRADS, and ACRTIRADS. Thereafter, we calculated the sensitivity, specificity, PPV, and NPV, along with the number of possible fine-needle aspiration (FNA) sparing, according to each NUS algorithm and to clustering risk classes within three macro-groups (low, intermediate, and high risk).

**Results:**

In a real-life setting of thyroid nodule management, available NUS scoring systems show good accuracy at ROC analysis (AUC up to 0.647) and higher NPV (up to 96%). The ability in FNA sparing ranges from 10 to 38% and reaches 44.2% of potential FNA economization in the low-risk macro-group. Considering our cohort, ACRTIRADS and AACE/ACE-AME scores provide the best compromise in terms of accuracy and spared cytology.

**Conclusions:**

Despite several limitations, available NUS do appear to assist physicians in clinical practice. In the context of a common disease, such as thyroid nodules, higher accuracy and NPV are desirable NUS features. Further improvements in NUS sensitivity and specificity are attainable future goals to optimize nodule management.

**Key Points:**

• *Thyroid nodule ultrasound scores do assist clinicians in real practice*.

• *Ultrasound scores reduce unnecessary diagnostic procedures, containing indolent thyroid microcarcinoma overdiagnosis*.

• *The variable malignancy risk of the “indeterminate” category negatively influences score’s performance in real-life management of thyroid lesions*.

**Supplementary Information:**

The online version contains supplementary material available at 10.1007/s00330-021-07703-5.

## Introduction

The progressive increase in detection of asymptomatic thyroid nodules is generating a relevant cost for thyroid diagnostic procedures [1]. As a consequence, the dramatic upsurge of newly diagnosed differentiated thyroid cancers (DTC) [2, 3] has become a tangible reality of endocrinology practice [4]. Nonetheless, the reported survival rate for DTC is more than 98% [5], meaning that, in the large majority of cases, the treatment of malignant nodules is unlikely to affect the overall prognosis [6]. In fact, since 2014, mortality has not substantially changed, due to the increase of microcarcinomas and of small DTC, to the aforementioned incidental finding in cervical US and to unjustified screening campaigns. Moreover, the probability of developing an invasive DTC is 0.6% and 1.8%, respectively, for male and female patients, and, among all the new diagnoses of DTC, the estimated specific death rate stands at 3.8% [7].

In order to contain overdiagnosis and unnecessary tests, several Scientific Societies of Endocrinologists and Radiologists have issued recommendations [1, 8–11] for a more cautious use of cytology in nodules without “suspect” features at US examination and for avoiding surgery in cases without clear signs of cytological malignancy. Notably, a number of different nodule ultrasound scores (NUS), also known under the general definition of TIRADS, have been proposed as a guidance tool for further diagnostic procedures in thyroid nodule disease [1, 8–11]. Although the development of those algorithms was based on the analysis of data collected in large clinical samples, parameters used for NUS differ across the different algorithms. Therefore, it is possible that the same nodule could be classified as “low risk” with one score and as “intermediate risk” with another one. Reasons for heterogeneity include the fact that different scores were developed and validated in different settings and populations that might be inhomogeneous for incidence of DTC and that might suffer from referral bias [1, 8–12].

A further problem is represented by the inherently low reproducibility of NUS [13, 14], which is inevitably an operator-dependent procedure. In addition, NUS scores do not consider simple clinical and demographic characteristics (such as gender and age) which affect the incidence of DTC in the general population [12, 15].

The aim of this cross-sectional study is to verify the suitability and the advantages in nodule management of five available NUS (KTIRADS, ATA, AACE/ACE-AME, EUTIRADS, ACRTIRADS) [1, 8–11]. Moreover, we retrospectively evaluated the potential ability in FNA sparing, linking NUS indications to the real practice of the Florence Endocrinology Outpatients Clinic.

## Materials and methods

The study was performed as a retrospective observational survey. Among all patients referred to our tertiary Endocrinology outpatient clinics for assessments of thyroid nodules between February 1, 2008, to February 1, 2018, we considered eligible all consecutive adult subjects (i.e., age > 18 years) for whom fine-needle aspiration (FNA) was indicated, and who provided a written informed consent. The real-life recommendation for a cytological examination was given combining several clinical and US parameters [4, 16], as summarized in Table 1 of the supplementary materials. Non-diagnostic cytology and nodules with clinical or incomplete US assessments were not included in this study. In addition, nodules with a size lower than 10 mm were also excluded from the analysis, considering that most of the available scores do not routinely recommend FNA for sub-centimeter thyroid nodules.

### Clinical and NUS assessments

Ultrasonographic examinations were performed with a conventional real-time scanner (ESAOTE Technos MP, MyLab™Twice, ESAOTE SPA©), equipped with a linear transducer operating at 10 MHz. All US examinations have been performed by the same endocrinologists (G.P., A.C., C.P., L.P.), experienced in neck US for more than 10 years. A full description of the thyroid gland and nodules was carried out, by filling in a standardized check-list, containing all the clinical information and US nodule features. Each nodule description included size (three-dimensional), composition (solid, mixed, or cystic), position of the solid portion in case of a mixed nodule (eccentric or not), echogenicity (anechoic, hyperechoic, or isoechoic, slightly hypoechoic, hypoechoic, or marked hypoechoic), halo (present, absent, or present but discontinuous or thick), margins (well defined or smooth, irregular or blurred), shape (taller than wider), presence of echogenic foci (hyperechoic spot, macro- and microcalcifications), rim calcification with extrusive soft tissue component, and type of vascularization (absence of flow signals; perinodular and absent or slight intranodular blood flow; marked intranodular blood flow or mixed) [17]. Elastography evaluation was not performed in all subjects, so this parameter has not been considered further.

### Cytological and histological assessments

Each FNA was performed by expert surgeons using capillary technique, under the guidance of the aforementioned endocrinologists experienced in neck US. Thin-layer slides were examined by two expert pathologists, who applied the cytological classification of the British Thyroid Association (BTA) [18], until May 2014, and, after that, of the Society for Anatomic Pathology and Cytology joined with the Italian Division of the International Academy of Pathology (SIAPEC-IAP) [19]. According to the SIAPEC-IAP classification [19], we categorized nodules as “negative cytology” nodules (with TIR 2 or TIR 3A in at least two consecutive samples), and “positive cytology” nodules (TIR 3B, TIR 4, TIR 5, with consequent surgical referral). According to BTA classification [18], all Thy3 responses obtained before 2014 were also categorized as “positive cytology” and potentially referred to surgery. An indeterminate category has a variable malignancy risk, notably after the adoption of the SIAPEC-IAP classification, which divided this class into two subgroups: TIR 3A (low-risk indeterminate lesion) and TIR 3B (high-risk indeterminate lesion), reflecting a different neoplastic risk and diagnostic taking over [20]. Because of that and the further bias added by changing cytological classification during the study, we also performed a second analysis. The latter considers only thyroid cytology from May 2014, when the new SIAPEC-IAP classification was adopted. In this case, we also excluded indeterminate cytology in order to improve the uniformity of the sample and to reduce a possible bias in the malignancy outcome. Final histology was staged according to TNM 2010 and 2017 [21, 22].

### Ultrasonographic scores

For valid cytology, we retrospectively and blindly applied five NUS (KTIRADS, ATA, AACE/ACE-AME, EUTIRADS, ACRTIRADS) [1, 8–11], assigning each nodule to its corresponding US class (Table 2, supplementary materials), working in not-fixed pairs of endocrinologists; in the event of disagreement about the NUS scoring, the other pair addressed the issue. Each score matches variable descriptive US features and nodule size, providing a stratification of the malignancy risk and indications for further diagnostic insights. We, thereafter, calculated the PPV and NPV of different NUS and the size of possible FNA sparing. Finally, we also performed the aforementioned analysis grouping similar NUS classes, according to the relative malignancy risk. Hence, we developed three macro-risk areas, i.e., low, intermediate, and high risk, considering as low-risk classes providing < 5% malignancy risk; as intermediate-risk classes between 5 and 20% risk, and as high-risk classes with > 20% risk. It is worthy to note that KTIRADS 4 class was included within the high-risk class, because of its broad interval of expected malignancy (15–50%), less consistent with an intermediate-risk category.

The ACRTIRADS classification [8] is the only one that assigns a score ranging from 0 to 3 points to each main ultrasound feature, the total score identifying the level of relative suspicion of the nodule.

The interobserver agreement was estimated considering a total sample of 250 thyroid nodules. Each operator performed a blind revision of frames from the same random cohort of thyroid lesions to classify each nodule according to the NUS scores investigated. Thereafter, we matched results to obtain the interobserver NUS variability.

### Statistical analysis

Data were expressed as mean ± SD when normally distributed and as median [quartiles] when non-normally distributed. The categorical variables were compared using chi-squared test. Sensitivity and specificity were calculated as the probability of finding or excluding positive cytology within each US category, respectively. NPV and PPV were calculated as the percentage of positive and negative cytology within each US category, respectively. NUS score accuracy was deduced by the area under the curve (AUC) of ROC curves. For NUS scoring within descriptive classes, the ROC curves were built by giving an increasing score ranging from 1 to 3 (AACE/ACE-AME), 1 to 4 (KTIRADS, EUTIRADS), and from 1 to 5 (ATA, ACRTIRADS). Considering that ACRTIRADS already provides a continuous scoring (range 0–14), these values were introduced as a continuous variable into ROC curves. Interobserver variability was calculated with Cohen’s *κ* statistics. The accordance rate was interpreted as follows: 0 to 0.20: slight; 0.21 to 0.40: fair; 0.41 to 0.60: moderate; 0.61 to 0.80: substantial; and 0.81 to 1.0: almost perfect agreement [23]. All statistical analyses were performed on SPSS for Windows 26.0.

## Results

**Fig. 1:**
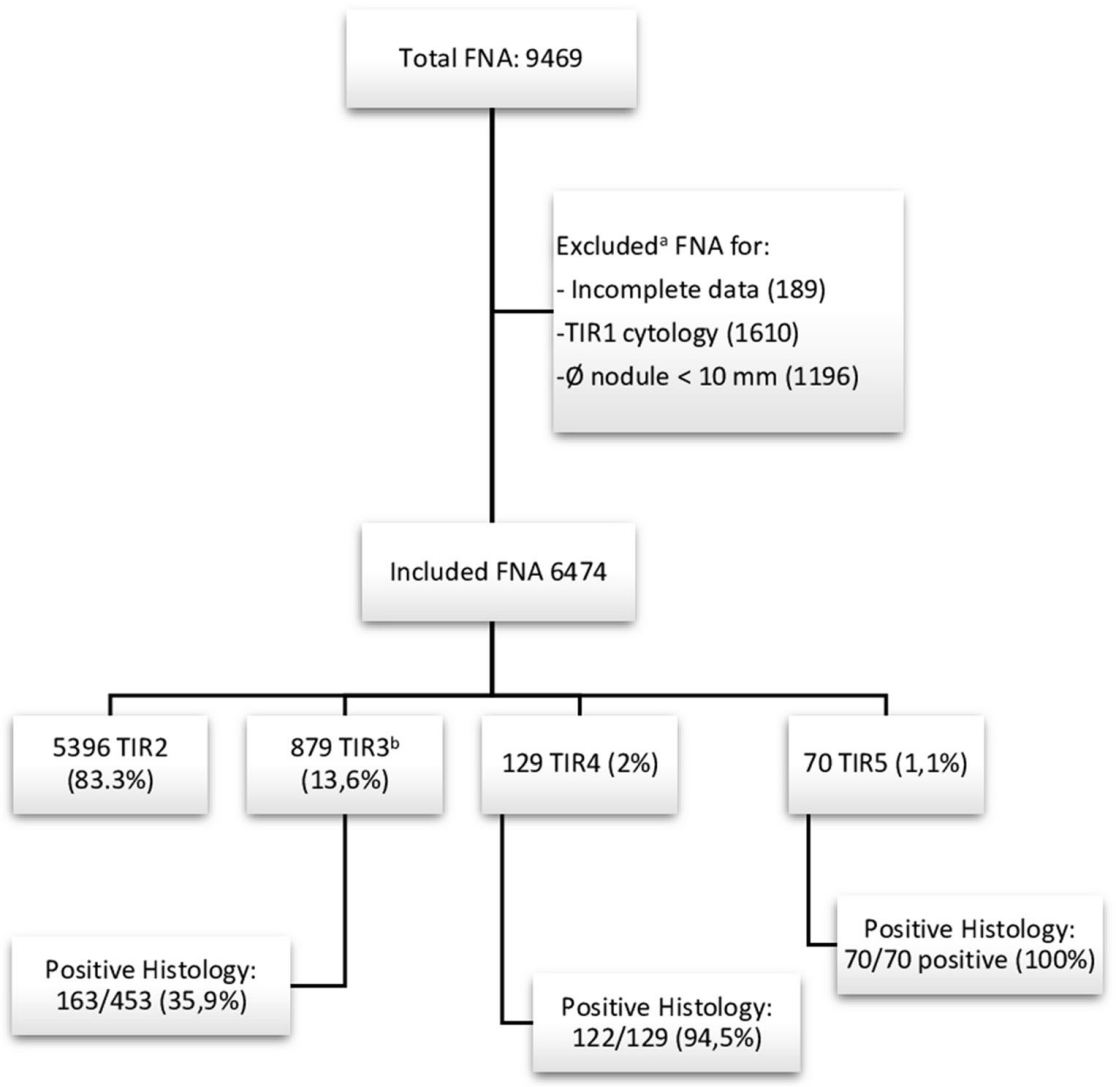
Flowchart of the study. ^**a**^Excluded for incomplete data or inconclusive cytology or sub-centimetric size (fine-needle aspiration is rarely indicated by any TIRADS for the latter). ^b^Indeterminate cytology: of those, 283 were classified as Thy3, according to the British Cytological classification (before 2014), and, later on, 596 ones as TIR3A (370) and TIR3B (226), according to the SIAPEC-IAP classification. *Ø* = size of the lesion

A flowchart of the present clinical sample is shown in Fig. 1. The cohort includes 6474 valid nodules from 6401 patients: 1402 males and 4999 females.

The cytological results and rate of positive histology are shown in Fig. 1. Through a combination of clinical, NUS, and cytological features, surgical referral was given to 708 subjects, according to the recommendations of International Societies [1, 4]. Of those, 509 nodules had an indeterminate cytology (283 Thy3 before 2014 and 226 TIR 3B after 2014), 129 TIR 4 and 70 TIR 5. Total thyroidectomy or lobectomy was performed in 652 subjects. The main histological types are summarized in Table 1.

**Table 1:** Main positive histology divided into well and poorly differentiated thyroid cancers and not thyroid cancer origin

	Histology	Number	Percent
Well-differentiated thyroid cancer	Papillary classic	166	46.8
	Follicular variant of papillary classic	97	27.3
	Medullary thyroid cancer	12	3.4
	Other histotype*	70	19.8
Poorly differentiated thyroid cancer	Insular cancer variant	3	0.8
	Other histotype**	5	1.4
Not thyroid cancer	Metastasis from other cancer	2	0.5
Total		355	100

*Follicular carcinoma, oncocytic variant, papillary or follicular variant of papillary with oxyphilous cells, Hurtle cell tumors, follicular non-invasive or minimally invasive and follicular with Hurtle cells

**Tall cell cancer, anaplastic, papillary with solid areas of dedifferentiation, papillary with areas of dedifferentiation and follicular cancer with Hurtle cell with low degree of differentiation

According to the US features, we matched each nodule to its corresponding score class within the investigated NUS score classifications (Table 2). Based on the achieved distribution in the various US categories, we assessed the proportion of pathological cytology within each score subgroup. Table 2 also shows results stratified according to the expected and to the observed FNA, performed according to clinical practice, where the cutoff size is not standardized. We also reported the proportion of FNA and related cytology that could be spared by following the relative NUS score suggestions. No difference was observed according to gender or other clinical features (not shown).

**Table 2:** Prevalence of malignancy for each class of the US scores, according to cytological outcome: KTIRADS, ATA, AACE/ACE-AME, EUTIRADS, ACRTIRADS

			Expected FNA				Observed FNA				Potential spared FNA			
	Class	Expected malignancy^c^, %	Size (mm)	Negative cytology^a^, % (no.)	Positive cytology^b^, % (no.)	Total FNA (no.)	Size (mm)	Negative cytology^a^, % (no.)	Positive cytology^b^, % (no.)	Total FNA (*N*)	Size (mm)	Negative cytology^a^, % (no.)	Positive cytology^b^, % (no.)	Total FNA (no.)
KTIRADS	KTIRADS2	< 3	≥ 20	97% (227)	3% (7)	234	-	95.3% (348)	4.7% (17)	365	-	92.4% (121)	7.6% (10)	131
	KTIRADS3	3–15	≥ 15	91.7% (2102)	8.3% (191)	2293	-	92.2% (2607)	7.8% (220)	2827	-	94.6% (505)	5.4% (29)	534
	KTIRADS4	15–50	≥ 10	88.1% (2381)	11.9% (323)	2704	≥ 10	88.1% (2381)	11.9% (323)	2704				
	KTIRADS5	> 60	≥ 10	74.2% (429)	25.8% (149)	578	≥ 10	74.2% (429)	25.8% (149)	578				
	**KTIRADS tot**					**5809**				**6474**				**665**
ATA	Benign	< 1	-	-	-	-	-	100% (53)	0% (0)	53	-	-	-	-
	Very low risk	< 3	≥ 20	100% (9)	0% (0)	9	-	100% (12)	0% (0)	12	-	100% (3)	0% (0)	3
	Low risk	5–10	≥ 15	92.3% (2681)	7.7% (225)	2906	-	92.6% (3319)	7.4% (266)	3585	-	94.0% (638)	6.0% (41)	679
	Intermediate risk	10-20	≥ 10	88.6% (1135)	11.4% (146)	1281	≥ 10	88.6% (1135)	11.4% (146)	1281				
	High risk	> 70–90	≥ 10	78.4% (851)	21.6% (234)	1085	≥ 10	78.4% (851)	21.6% (234)	1085				
	**ATA Tot**					**5281**				6016*				**682**
AACE/ACE-AME	Low risk	< 1	> 20	93.2% (828)	6.8% (60)	888	-	93.5%(1481)	6.5% (103)	1584	-	93.8% (653)	6.2% (43)	696
	Intermediate risk	5-20%	> 20	90.6% (1405)	9.4% (145)	1550	≥ 10	90.8% (3016)	9.2% (306)	3322	-	90.9% (1611)	9.1% (161)	1772
	High risk	50–90%	≥ 10	80.9% (1268)	19.1% (300)	1568	≥ 10	80.9% (1268)	19.1% (300)	1568	-			
	**AACE Tot**					**4006**				**6474**				**2468**
EUTIRADS	EUTIRADS 2	0	-	100% (8)	0% (0)	8	-	100% (57)	0% (0)	57	-	100% (8)	0% (0)	49
	EUTIRADS 3	2–4%	> 20	91.8% (1054)	8.2% (94)	1148	-	91.6% (1760)	8.4% (161)	1921	-	91.4% (706)	8.7% (67)	773
	EUTIRADS 4	6–17%	> 15	91.8% (1991)	8.2% (179)	2170	≥ 10	91.5% (2680)	8.5% (248)	2928	-	90.9% (689)	9.1% (69)	758
	EUTIRADS 5	26–87%	> 10	81.4% (1194)	18.6% (272)	1466	≥ 10	80.9% (1268)	19.1% (300)	1568	-	72.5% (74)	27.4% (28)	102
	**EUTIRADS Tot**					**4792**				**6474**				**1682**
ACRTIRADS	ACRTIRADS 1	< 2%	-	100% (8)	0% (0)	8	-	100% (19)	0% (0)	19		100% (11)	0% (0)	11
	ACRTIRADS 2	< 2%	-	100% (18)	0% (0)	18	-	98.7% (226)	1.3% (3)	229		99.1% (208)	1.4% (3)	211
	ACRTIRADS 3	5%	≥ 25	92.2% (166)	7.8% (14)	180	-	94.4% (571)	5.6% (34)	605		95.3% (405)	4.7% (20)	425
	ACRTIRADS 4	5-20	≥ 15	91.0% (2472)	9.0 % (245)	2717	≥ 10	91.1% (3093)	8.9% (301)	3394		91.7% (621)	8.3 % (56)	667
	ACRTIRADS 5	> 20	≥ 10	83.3% (1856)	16.7% (193)	2227	≥ 10	83.3% (1856)	16.7% (193)	2227				
	**ACRTIRADS Tot**					**5150**				**6474**				**1324**

^a^According to SIAPEC-IAP classification, TIR 2 or TIR 3A in at least two consecutive samples were considered as negative cytology, while TIR 3B, TIR 4, and TIR 5 as positive cytology

^b^(With surgical referral). According to British Thyroid Association, all Thy3 obtained before 2014 were also categorized as positive cytology and potentially referred to surgery

^c^Expected malignancy according to each US score class

*For 458 nodules, the ATA score was not applicable

Concerning benign or very low–risk nodules (attended malignancy < 3%), present results are essentially in line with the majority of NUS algorithms. In contrast, KTIRADS2 and AACE/ACE-AME “low risk” underestimated cytological outcomes, at 4.7% and 6.5%, respectively. Concerning the high-risk classes, cytological results suggest that there is a systematic overestimation of the real risk of a positive cytology, with the lowest overestimation for EUTIRADS5 and ACRTIRADS5. Positive cytology in low–intermediate classes (those with expected malignancy ranging from 5 to 20%) variably recapitulates predicted risks, with a substantial concordance with the expected malignancy. In our analysis, we were unable to classify 458 nodules (7% of all population) according to ATA score [1], because some NUS findings (i.e., isoechoic nodules with irregular margins or microcalcifications; mixed nodules with doubtful eccentric solid portion) could not be allocated to any of the official ATA classes; consequently, these lesions were excluded from the main analysis for ATA classification. Among the unclassifiable ATA nodules, 13.8% had a positive cytology.

Table 3 supplementary materials shows a second sub-analysis of the present sample (considering only thyroid cytology from May 2014 using SIAPEC-IAP classification [19] and excluding “Intermediate” cytology) conducted on a smaller sample of 2547 cytology cases. Overall, we found an improved performance of low risk classes towards expected malignancy although there is a general underestimation of the remaining categories.

Sensitivity, specificity, NPV, and PPV for the different NUS algorithms for positive cytology are summarized in Table 3, along with the proportion of potentially spared FNA. Sensitivity with different NUS scores ranges from 50.1% (AACE/ACE-AME) to 94.5% (KTIRADS), whereas specificity ranges between 14.8 and 50.3%. Moreover, we found very high NPV, from 89.9 to 95.6% for all NUS scores. In contrast, PPV is around 11% or less (Table 3). The same analysis on macro-risk areas is shown in Table 4 and provides similar results, with very high NPV and satisfying sensitivity, but poor PPV and underwhelming specificity. The rate of potentially spared FNA was calculated according to the difference between observed and expected FNA results (Table 2). This share corresponds to the number of nodules that should not be further investigated, according to a combination of size and NUS features. In most cases, the proportion of potentially spared FNA is interesting, reaching 38.1% with the AACE/ACE-AME score (Table 3). Considering the proportion spared for macro-risk classes, we found an interesting potential difference in the lower risk classes, up to 44.2% (Table 4). Among them, considering the positive cytology of the potentially spared FNA subgroup referred to surgery, we found a high rate (up to about 90% of cases) of low stages of papillary thyroid cancer (pT1apNx and pT1bpNx). Finally, in order to verify the impact of the ATA unclassified nodules, we also performed a sensitivity analysis, by excluding the 458 nodules from the whole population. Results are shown in Tables 4 and 5 in supplementary materials. The sensitivity analysis did not show any consistent variation in the final results.

**Table 3:** Sensitivity, specificity, positive predictive value (PPV), negative predictive value (NPV) for each ultrasound score, and hypothetical percentage of spared FNA depending on each score recommendations

US score	Total FNA	Sensitivity	Specificity	PPV (CI 95%)	NPV (CI 95%)	Spared FNA (%)
KTIRADS	6474	94.5%	14.8%	11.5% (± 0.8%; 10.7–12.3%)	95.6% (± 1.3%; 94.3–96.9%)	665 (10.3%)
ATA	6016	84.5%	19.5%	7.7% (± 0.3%; 7.4–8%)	94.1% (± 1.7%; 92.4–95.8%)	682 (11.3%)
AACE/ACE-AME	6474	50.1%	50.3%	8.4% (± 2.7%; 5.7–11.1%)	91.7% (± 0.8%; 90.9–92.5%)	2468 (38.1%)
EUTIRADS	6474	80.6%	20.7%	10.8% (± 2%; 8.8–12.8%)	89.9% (± 0.7%; 89.2–90.6%)	1682 (25.9%)
ACRTIRADS	6474	72.7%	31.7%	8.9% (± 1%; 7.9–9.9%)	93.0% (± 1.3%; 91.7–94.3%)	1324 (20.6%)

**Table 4:** Sensitivity, specificity, PPV, NPV, and hypothetical percentage of spared FNA, grouping comparable ultrasound score classes

Risk class (expected malignancy risk)	US score classes	Sensitivity (CI 95%)	Specificity (CI 95%)	PPV (CI 95%)	NPV (CI 95%)	% spared FNA
Low-risk classes, < 5%	KTIRADS 2	57.9% (± 5.6%; 52.3–63.5%)	44.3% (± 1.6%; 42.7–45.9%)	7.3% (± 1.0%; 6.3–8.3%)	93.3% (± 1.1%; 92.2–94.4%)	44.2%
	ATA benign–very low risk					
	AACE low					
	EUTIRADS 2–3					
	ACRTIRADS 1–2					
Intermediate-risk classes, 5–20%	KTIRADS3	75.3% (± 2.0%; 73.3–77.3%)	27.2% (± 0.7%; 26.5–27.9%)	8.7% (± 0.4%; 8.3–9.1%)	92.2% (± 0.7%; 91.5–92.9%)	26.9%
	ATA low–intermediate					
	AACE intermediate					
	EUTIRADS 4					
	ACRTIRADS 3–4					
High-risk classes, > 20%	KTIRADS 4–5	98.1% (± 0.7%; 97.4–98.8%)	0.9% (± 0.2%; 0.7–1.1%)	15.7% (± 0.7%; 15.0–16.4%)	72.5% (± 2.4%; 70.1–74.9%)	1.1%
	ATA high					
	AACE high					
	EUTIRADS 5					
	ACRTIRADS 5					

**Fig. 2:**
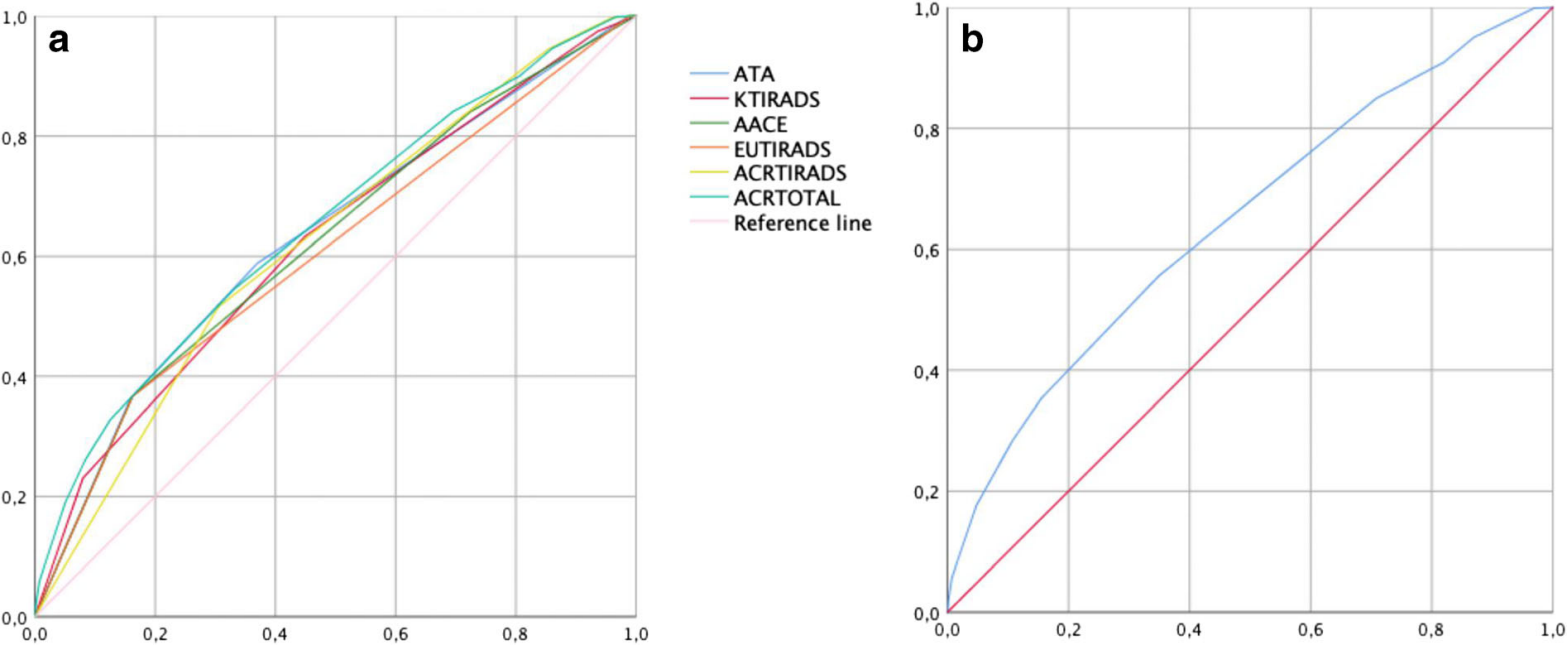
**a** ROC curves according to positive cytology for all NUS scores. The area under ROC curves are 0.623 (CI 95%; 0.599–0.647) for KTIRADS; 0.632 (CI 95%; range 0.608–0.656) for ATA; 0.623 (CI 95%; 0.599–0.646) for AACE/ACE-AME; 0.606 (CI 95%; 0.582–0.631) for EUTIRADS; 0.622 (CI 95%; 0.600–0.645) for ACRTIRADS range 1–5. **b** ROC curve considering the total scoring of ACRTIRADS (points 1–14). The area under ROC curve is 0.647 (CI 95%; 0.625–0.669)

Score accuracy, calculated through ROC curve analysis, is shown in Fig. 2, where the whole population sample was considered. Notably, the best NUS accuracy was obtained with ACRTIRADS total scoring (ranging from 1 to 14): 0.647 (CI 95%; 0.625–0.669) (Fig. 2b). The same analysis—performed considering only positive cytology from May 2014 and excluding intermediate cytology—is shown in Fig. 1 of supplementary materials revealing an overall improved NUS accuracy.

The interobserver agreement for each NUS score was determined in a sample of 250 nodules. Results are shown in Table 6 of supplementary materials. Cohen’s *κ* analysis indicates a concordance from moderate to substantial in every NUS scoring (0.50–0.73).

## Discussion

Based on the present results, despite differences in each algorithm’s design, available NUS systems show satisfying performance in terms of accuracy, and provide useful information in avoiding unnecessary FNA in the real-life management of thyroid nodules, up to almost 40%. Although the single-center, retrospective design of the study limits its widespread validity, our results reflect everyday clinical practice, incorporating also “indeterminate” cytology, a key departure from other series, where this category has been almost systematically excluded [12, 24–26].

In recent years, many associations of endocrinologists and radiologists have provided several US scoring systems [1, 8–11], based on sets of US features and nodule size, in order to allow for a more rational and uniform management of thyroid lesions. The purpose of these classifications is not only to identify cases of cancer but also to correctly address the diagnostic process, reducing unnecessary procedures. Such indications are based on results from several surveys, although specific validation for some of them (ATA [1], AACE/ACE- AME [9]) has not yet been provided. Other scoring systems were validated, but only in particular settings, i.e., excluding indeterminate cytology [24, 25].

The present study analyzes five of the major NUS algorithms, verifying their potential clinical impact by comparing the expected risk of malignancy based on different NUS with their relative cytological outcomes. We essentially found a mild overestimation in the lower risk classes and a consistent underestimation in the high-risk ones. This distortion could be partially explained by the broad sample size, which provides many negative cytological results, together with the wide proportion of indeterminate cytology. Notably, the exclusion of this last category appreciably improves NUS diagnostic accuracy also in our analysis, but at the price of a substantial underestimation of all risk classes. Moreover, another accepted adjustment can be seen in the assumption of TIR 2 cytology as a final negative histological result, because, by definition, these patients do not undergo surgery. Such arbitrary choices could improve the apparent score’s performance, but are conceptually wrong, since they do not correspond to real-life practice. In fact, TIR 2 cytology still bear a small potential of incertitude [27, 28], in particular in large size nodules. In addition, indeterminate cytology represents a consistent proportion of cytological results. However, concerning high-risk classes, our results are at odds with those of a recent report in a smaller series of patients [15], despite similarities in the clinical setting in which the patients were enrolled. This fact points to potential differences due to minor heterogeneities in case mix and/or clinical procedures.

From a clinical perspective, it is important to know the number of potentially spared FNA by applying the different NUS algorithms. After stratifying cytological results as dummy variables according to the possible need for surgical consideration, all NUS algorithms showed a good sensitivity and a very high NPV, but a poor specificity and PPV at cytology, even if fairly consistent with a previous study [29]. Notably, NPV and sensitivity are the most useful parameters to manage a widespread disease with a very high benignity rate, such as thyroid nodules. Moreover, the rate of possibly spared FNA, although variable, appears significant in real practice, especially for low-risk classes of all NUS, which represent the largest proportion of thyroid lesions. Additionally, as found in other studies [e.g., 12, 25], a portion of the nodule population might not be properly allocated within ATA classes; however, in the present study, the share of ATA unclassified nodules resulted as being very small and, even excluding those nodules from the whole cohort in a sensitivity analysis, we did not observe substantial changes, in particular in FNA sparing. Finally, the share of DTC diagnosis virtually lost within the spared FNA is represented by very low stage malignancies, whose delayed diagnosis would not affect patients’ prognosis. This is tantamount to say that NUS reduce unnecessary FNA and consequently overdiagnosis. This fact is also confirmed by a recent meta-analysis, which explored the ability of the same five NUS to select thyroid nodules warranting FNA [29]. In that study [29], ACRTIRADS algorithm showed the best performance. In their conclusion, the authors highlight the point of a general limitation in comparing ultrasound scores because of several clinical and methodological biases. Moreover, most NUS were conceived to identify papillary thyroid cancer, limiting the score performance in other cancer histotypes (i.e., follicular cancer, which usually appears as an isoechoic nodule) [29].

Considering our results, ROC curve analysis suggests that all the NUS scoring algorithms show virtually similar accuracy, although numerically better results were obtained by ACRTIRADS [8]. In fact, ACRTIRADS [8] shows the highest AUC, when the total points scoring system was considered, while, among descriptive NUS, ATA algorithm [1] shows the best accuracy.

Concerning the ability of sparing FNA, AACE/ACE-AME, EUTIRADS, and ACRTIRADS [8, 9, 11] provides a favorable rate of spared FNA, which represents a suitable goal in clinical practice. In fact, more than one third of cytology could be avoided with AACE/ACE-AME classification [9], with a good specificity, but at the expense of a poorer sensitivity. On the other hand, EUTIRADS and ACRTIRADS [8, 11] are able to reduce FNA by one in four and one in five, respectively, preserving better sensibility. Finally, despite the accuracy of ATA classification [1], according to the population sample, this algorithm shows variable proportions of unclassified nodules, resulting as less effective in reducing the number of spared cytology. For these reasons, we can conclude that, in our population, the best compromise in FNA sparing ability and accuracy is provided by AACE/ACE-AME and ACRTIRADS [8, 9]. Those classifications allow a suitable allocation of thyroid lesions to the appropriate classes, improving nodule selection and FNA sparing ability. Moreover, thanks to its points system design, the ACRTIRADS score [8] appears easy to handle and might be appealing for untrained US operators. On the other hand, the AACE/ACE-AME [9] concise structure simplifies nodules classifications, reducing the possible distortion in class allocation.

Our study presents some relevant limitations. First, it is a retrospective analysis; second, it is based on a population from a single tertiary hospital, with an evident selection bias. Furthermore, in the real world, the recommendation for FNA relies not only on thyroid nodule US features but also on clinical factors.

On the other hand, some important strengths should be recognized: we analyzed a large sample, for which we systematically collected all ultrasound features and clinical information. In addition, the same population has been examined by the same experienced endocrinology (G.P., A.C., C.P., L.P.) and pathology team, over the years. The reliable NUS agreement of the operators further supports our outcomes, as already shown in other series [13, 14]. Finally, our study really reflects clinical practice on a wide and variable population, where it is not always possible to apply a strict, standardized medical strategy.

In conclusion, NUS may be deemed as a worthy ally to all physicians in a real-life setting. However, the large number of available classifications, the lack of multicenter validation or prospective studies with a centralized laboratory, the variety in study designs, and some discrepancies among NUS classes still represent the current limits of these tools. Moreover, a further, better allocation within risk classes is advisable, in order to reduce such heterogeneity and potential misdiagnosis. Despite some relevant structural frailties, the achievement of a good compromise in terms of NUS sensitivity and specificity is a realistic clinical goal, notably in well-established long-standing work teams. Our experience has provided evidence, in favor of NUS adoption, with significant clinical benefits, achievable mostly with ACRTIRADS and AACE/ACE-AME scores [8, 9] in our institution. The share of spared cytology and the level of accuracy for each NUS may act as effective indicators of a score’s performance, driving physicians’ teams towards the best US algorithm. Hence, according to patient population and operator inclinations, the adoption of the most fitting NUS by each hospital might be desirable to harmonize ultrasonographic descriptions and diagnostic procedure indications.

## Supplementary Information

ESM 1 (DOCX 309 kb)

## Abbreviations

BTA: British Thyroid Association
DTC: Differentiated thyroid cancer
FNA: Fine-needle aspiration
NUS: Nodule ultrasound scores
SIAPEC-IAP: Society for Anatomic Pathology and Cytology joined with the Italian Division of the International Academy of Pathology
