# Supplementary material for: Choosing the best algorithm among five thyroid nodule ultrasound scores: from performance to cytology sparing—a single-center retrospective study in a large cohort
Source: Eur Radiol. 2021 Feb 18;31(8):5689–98. doi: 10.1007/s00330-021-07703-5 (PMC8270877; doi:10.1007/s00330-021-07703-5)
Supplement: Supplementary file 1 — (DOCX 309 kb) [file 330_2021_7703_MOESM1_ESM.docx]

**Table 1. Suspect clinical and US features considered in real-practice to provide FNA indication for thyroid nodules.**

| **Indication to FNA** |
| --- |
| **Suspect clinical factors**   - Neck irradiation - New onset of palpatory nodule and/or presence of suspect clinical features (firmness, hoarseness, dysphagia) - Fluorodeoxyglucose (FDG)-positron emission tomography (PET) uptake - Familiarity for thyroid cancer - Male gender |
| **Suspect Ultrasonographic features**   - Solid or mainly solid nodules with at least one feature among:   Hypoechogenity  Microcalcification or hyperechogenic spots  Irregular or spiculate margins  Extracapsular extrusion   - Regional lymphadenopathy - Rapid US evolution during follow-up (i.e. volume increase during less than 12 months) |

**Table 2. Panel 2A Main feature of five ultrasonography scores with expected malignancy risk for each score class. Panel 2B ACR TIRADS scoring panel.**

**Panel 2A**

|  | N° | Level of suspicion | US Nodule description | ROM % | ∅ TO FNA (mm) |
| --- | --- | --- | --- | --- | --- |
| KTIRADS | **1** | Normal | No Nodules | - | - |
|  | **2** | Benign | Spongiform  Pure cyst or partially cystic with comet tail artifact** | <3 | ≥20 |
|  | **3** | Low | Partially cystic or isohyperechoic nodule without any of 3 supicious US features | 3-15 | ≥15 |
|  | **4** | Intermediate | Solid hypoechoic nodule without any of 3 supicious US features  Partially cystic or isohyperechoic with any of 3 suspicious features | 15-50 | ≥10 |
|  | **5** | High | Solid hypoechoic with any of US suspect features:   - Spiculated/microlobulated margins - Non-parallel orientation - Microcalcification | >60 | ≥10 |
|  |  |  |  |  |  |
| ATA |  | Benign | Purly cystic (no solid component) | <1 | No |
|  |  | Very Low | Spongiform or partially cystic nodules, without US features of low, intermediate, high | <3 | ≥20 |
|  |  | Low | Isoechoic or hyperechoic solide nodules, or partially cystic nodules with eccentric solid areas, without US suspect features | 5-10 | ≥15 |
|  |  | Intermediate | Hypoechoic solid nodule with smooth margins, without US suspect features | 10-20 | ≥10 |
|  |  | High | Solid hypoechoic nodule or solid hypoechoic component of partial cystic nodule with one or more US suspect features:   - Irregular margins - Microcalcification - Taller than wide shape - Rim calcification with small extrusive soft tissue component - ETE (Evidence of Extra-Thyroidal Extention) | >70-90 | ≥10 |
|  |  |  |  |  |  |
| EUTIRADS | **1** | Normal | No nodules | - | - |
|  | **2** | Benign | Pure cyst;  Entirely spongiform | 0 | NO |
|  | **3** | Low | Ovoid, smooth isoechoic/hyperechoic;  no features of high suspicion | 2-4 | >20 |
|  | **4** | Intermediate | Ovoid, smooth, mildly hypoechoic;  no features of high suspicion | 6-17 | >15 |
|  | **5** | High | At least one of high US features:   - Irregular shape - Irregular margins - Microcalcifications   Marked hypoechogenicity (and solid) | 26-87 | >10 |
|  |  |  |  |  |  |
| AACE/ACE-AME |  | Low | Cyst or mostly cystic nodules with reverberating artifacts, and without US suspect features  Isoechoic spongiform nodules, either confluent or with regular halo | <1 | >20 |
|  |  | Intermediate | Slightly hypoechoic or isoechoic nodules, with ovoid-to-round shape, smooth or ill-defined margins. Maybe present:   - Intranodular vascularization - Elevated stiffness at elastography - Macro or continuous rim calcification - Indeterminate hyperechoic spots | 5-20 | >20 |
|  |  | High | Nodules with at least one of the following features:   - Marked hypoechogenicity - Spiculated or lobulated margins - Microcalcifications - Taller than wide shape (AP>TR) - Extrathyroidal growth - Patological adenopathy | 50-90 | >10 |
|  |  |  |  |  |  |
| ACR TIRADS*** | **1** | Benign | Total Score = 0 (TR1)*** | <2 | No |
|  | **2** | Not Suspicious | Total Score = 2 (TR2)*** | <2 | No |
|  | **3** | Mildly Suspicious | Total Score = 3 (TR3)*** | 5 | ≥25 |
|  | **4** | Moderately Suspicious | Total Score = 4-6 (TR4)*** | 5-20 | ≥15 |
|  | **5** | Highly Suspicious | Total Score = ≥7 (TR5)*** | ≥20 | ≥10 |

* ROM= risk of malignancy; **ROM < 1%; FNA, fine-needle aspiration; ***See ACR TIRADS scoring panel.

∅ = Diameter of lesion for which FNA is desirable

**Panel 2B.**

|  | Ultrasound features | | | | |
| --- | --- | --- | --- | --- | --- |
| Point | **Composition** | **Echogenicity** | **Shape** | **Margin** | **Echogenic Foci** |
| 0 | Cystic or almost completely cystic  Spongiform | Anechoic | Wider-than-taller | Smooth  Ill-defined | None or large comet-tail artefacts |
| 1 | Mixed cystic and solid | Hyperechoic or Isoechoic | - | - | Macrocalcification |
| 2 | Solid or almost completely solid | Hypoechoic | - | Lobulated or Irregular | Peripheral rim calcification |
| 3 | - | Very hypoechoic | Taller-than-wide | Extra-thyroidal extension | Punctate echogenic foci |

Reported cancer risk class of different ultrasound scores: KTIRADS, ATA, AACE/ACE-AME, EUTIRADS, ACR TIRADS

**Table 3- Prevalence of malignancy for each class of the US scores, according to cytology: KTIRADS, ATA, AACE/ACE-AME, EUTIRADS, ACR TIRADS.** The cohort on study was considered from May 2014, after the introduction of the new SIAPEC-IAP classification, and excluded indeterminate cytology TIR3A and TIR3B. ^a^According to SIAPEC-IAP classification, TIR 2 were considered as negative cytology, while TIR 4, TIR 5 as positive cytology ^b^ (with surgical referral). ^C^ Expected malignancy for each ultrasonographic score class.

|  |  |  | Potential FNA | | | | Real FNA | | | |
| --- | --- | --- | --- | --- | --- | --- | --- | --- | --- | --- |
|  | **CLASS** | **EXPECTED MALIGNANCY^c^**  **%** | **SIZE**  **(mm)** | **NEGATIVE CYTOLOGY^a^**  **%(N°)** | **POSITIVE CYTOLOGY^b^**  **%(N°)** | **Total FNA**  **(N°)** | **SIZE**  **(mm)** | **NEGATIVE CYTOLOGY^a^**  **%(N°)** | **POSITIVE CYTOLOGY^b^**  **%(N°)** | **Total FNA**  **(N)** |
| KTIRADS | KTIRADS2 | <3 | >=20 | 100% (59) | 0% (0) | 59 | - | 100% (91) | 0% (0) | 91 |
|  | KTIRADS3 | 3-15 | >=15 | 98.7%(882) | 1.3% (12) | 894 | - | 98.6%(1096) | 1.4% (16) | 1112 |
|  | KTIRADS4 | 15-50 | >=10 | 95.0%(1064) | 5.0%(56) | 1120 | >=10 | 95.0%(1064) | 5.0%(56) | 1120 |
|  | KTIRADS5 | >60 | >=10 | 80.4% (180) | 19.6%(44) | 224 | >=10 | 80.4% (180) | 19.6%(44) | 224 |
|  | **KTIRADS Tot** |  | | | | **2297** |  | | | **2547** |
|  | | | | | | | | | | |
| ATA | BENIGN | <1 | - | 100% (9) | 0% (0) | 9 | >=10 | 100% (14) | 0% (0) | 14 |
|  | VERY LOW RISK | <3 | >=20 | 100% (3) | 0% (0) | 3 | >=10 | 100% (3) | 0% (0) | 3 |
|  | LOW RISK | 5-10 | >=15 | 98.8%(1140) | 1.2% (14) | 1154 | >=10 | 98.7%(1415) | 1.3% (19) | 1434 |
|  | INTERMEDIATE RISK | 10-20 | >=10 | 95.1%(523) | 4.9%(27) | 550 | >=10 | 95.1%(523) | 4.9%(27) | 550 |
|  | HIGH RISK | >70-90 | >=10 | 87.3% (339) | 16.3%(66) | 405 | >=10 | 83.7% (339) | 16.3%(66) | 405 |
|  | **ATA Tot** |  | | | | **2121** |  | | | **2406*** |
|  | | | | | | | | | | |
| AACE/ACE-AME | LOW RISK | <1 | >20 | 99.7% (326) | 0.3% (1) | 327 | >=10 | 99.8%(559) | 0.2% (1) | 560 |
|  | INTERMEDIATE RISK | 5-20% | >20 | 97.7%(645) | 2.3% (15) | 660 | >=10 | 96.9%(1390) | 3.1% (45) | 1435 |
|  | HIGH RISK | 50-90% | >10 | 89.9% (286) | 10.1% (32) | 318 | >=10 | 87.3%(482) | 12.7% (70) | 552 |
|  | **AACE Tot** |  | | | | **1305** |  | | | **2547** |
|  | | | | | | | | | | |
| EUTIRADS | EUTIRADS 2 | 0 | - | 100% (8) | 0% (0) | 8 | >=10 | 100% (15) | 0% (0) | 15 |
|  | EUTIRADS 3 | 2-4% | >20 | 98.4% (371) | 1.6% (6) | 377 | >=10 | 98.8%(649) | 1.2% (8) | 657 |
|  | EUTIRADS 4 | 6-17% | >15 | 98.1%(878) | 1.9% (17) | 895 | >=10 | 97.1%(1285) | 2.9% (38) | 1323 |
|  | EUTIRADS 5 | 26-87% | >10 | 89.9% (286) | 10.1% (32) | 318 | >=10 | 87.3%(482) | 12.7% (70) | 552 |
|  | **EUTIRADS Tot** |  | | | | **1598** |  | | | **2547** |
|  | | | | | | | | | | |
| ACRTIRADS | ACRTIRADS 1 | <2% | - | - | - | - | >=10 | 100% (1) | 0% (0) | 1 |
|  | ACRTIRADS 2 | <2% | - | 100% (18) | 0% (0) | 18 | >=10 | 100% (58) | 0% (0) | 58 |
|  | ACRTIRADS 3 | 5% | >=25 | 96.4% (27) | 3.6% (1) | 28 | >=10 | 98.3% (114) | 1.7% (2) | 116 |
|  | ACRTIRADS 4 | 5-20 | >=15 | 98.8%(1144) | 1.2 % (14) | 1158 | >=10 | 98.4%(1436) | 1.6% (23) | 1459 |
|  | ACRTIRADS 5 | >20 | >=10 | 90.0%(822) | 10.0% (91) | 913 | >=10 | 90.0%(822) | 10.0% (91) | 913 |
|  | **ACRTIRADS Tot** |  | | | | **2117** |  | | | **2547** |

*For 141 nodules the ATA score was not applicable

**Table 4. Prevalence of malignancy for each class of the US scores, according to cytological outcome: KTIRADS, ATA, AACE/ACE-AME, EUTIRADS, ACR TIRADS. This analysis did not consider 458 ATA unclassified nodules for all NUS.** ^a^According to SIAPEC-IAP classification, TIR 2 or TIR 3A in at least two consecutive samples were considered as negative cytology, while TIR 3B, TIR 4, TIR 5 as positive cytology ^b^ (with surgical referral). According to British Thyroid Association, all Thy3 obtained before 2014 were also categorized as positive cytology and potentially referred to surgery. ^C^ Expected malignancy according to each US score class**.**

|  |  |  | Expected FNA | | | | Observed FNA | | | | Potential spared FNA | | | | |
| --- | --- | --- | --- | --- | --- | --- | --- | --- | --- | --- | --- | --- | --- | --- | --- |
|  | **CLASS** | **EXPECTED MALIGNANCY^c^**  **%** | **SIZE**  **(mm)** | **NEGATIVE CYTOLOGY^a^**  **%(N°)** | **POSITIVE CYTOLOGY^b^**  **%(N°)** | **Total FNA**  **(N°)** | **SIZE**  **(mm)** | **NEGATIVE CYTOLOGY^a^**  **%(N°)** | **POSITIVE CYTOLOGY^b^**  **%(N°)** | **Total FNA**  **(N)** | **SIZE**  **(mm)** | **NEGATIVE CYTOLOGY^a^**  **%(N°)** | | **POSITIVE CYTOLOGY^b^**  **%(N°)** | **Total FNA**  **(N°)** |
| KTIRADS | KTIRADS2 | <3 | >=20 | 97% (227) | 3% (7) | 234 | - | 95.3% (348) | 4.7% (17) | 365 | - | 92.4% (121) | | 7.6% (10) | 131 |
|  | KTIRADS3 | 3-15 | >=15 | 91.7%(2102) | 8.3% (191) | 2293 | - | 92.2%(2607) | 7.8% (220) | 2827 | - | 94.6%(505) | | 5.4% (29) | 534 |
|  | KTIRADS4 | 15-50 | >=10 | 88.4% (1986) | 11.6% (260) | 2246 | >=10 | 88.4% (1986) | 11.6% (260) | 2246 |  |  | |  |  |
|  | KTIRADS5 | >60 | >=10 | 74.2% (429) | 25.8% (149) | 578 | >=10 | 74.2% (429) | 25.8%(149) | 578 |  |  | |  |  |
|  | **KTIRADS Tot** |  | | | | **5351** |  | | | **6016** |  |  | |  | **665** |
|  | | | | | | | | | | |  | | | | |
| ATA | BENIGN | <1 | - | - | - | - | - | 100% (53) | 0% (0) | 53 | - | - | - | | - |
|  | VERY LOW RISK | <3 | >=20 | 100% (9) | 0% (0) | 9 | - | 100% (12) | 0% (0) | 12 | - | 100% (3) | 0% (0) | | 3 |
|  | LOW RISK | 5-10 | >=15 | 92.3%(2681) | 7.7% (225) | 2906 | - | 92.6%(3319) | 7.4% (266) | 3585 | - | 94.0%(638) | 6.0% (41) | | 679 |
|  | INTERMEDIATE RISK | 10-20 | >=10 | 88.6%(1135) | 11.4%(146) | 1281 | >=10 | 88.6%(1135) | 11.4%(146) | 1281 |  |  |  | |  |
|  | HIGH RISK | >70-90 | >=10 | 78.4% (851) | 21.6%(234) | 1085 | >=10 | 78.4% (851) | 21.6%(234) | 1085 |  |  |  | |  |
|  | **ATA Tot** |  | | | | **5281** |  | | | **6016** |  | | | | **682** |
|  | | | | | | | | | | |  | | | | |
| AACE/ACE-AME | LOW RISK | <1 | >20 | 93.2% (828) | 6.8% (60) | 888 | - | 93.5%(1481) | 6.5% (103) | 1584 | - | 93.8% (653) | 6.2% (43) | | 696 |
|  | INTERMEDIATE RISK | 5-20% | >20 | 90.6%(1405) | 9.4% (145) | 1550 | >=10 | 90.8%(3016) | 9.2% (306) | 3322 | - | 90.9%(1611) | 9.1% (161) | | 1772 |
|  | HIGH RISK | 50-90% | >=10 | 78.6% (873) | 21.4% (237) | 1110 | >=10 | 78.6% (873) | 21.4% (237) | 1110 | - |  |  | |  |
|  | **AACE Tot** |  | | | | **3548** |  | | | **6016** |  | | | | **2468** |
|  | | | | | | | | | | |  | | | | |
| EUTIRADS | EUTIRADS 2 | 0 | - | 100% (8) | 0% (0) | 8 | - | 100% (57) | 0% (0) | 57 | - | 100% (49) | 0% (0) | | 49 |
|  | EUTIRADS 3 | 2-4% | >20 | 91.8% (1054) | 8.2% (94) | 1148 | - | 91.6%(1760) | 8.4% (161) | 1921 | - | 91.3% (706) | 8.7% (67) | | 773 |
|  | EUTIRADS 4 | 6-17% | >15 | 91.7% (1990) | 8.3% (180) | 2170 | >=10 | 91.5%(2680) | 8.5% (248) | 2928 | - | 91.0% (690) | 9.0% (68) | | 758 |
|  | EUTIRADS 5 | 26-87% | >10 | 79.1% (814) | 20.9% (215) | 1029 | >=10 | 78.6% (873) | 21.4% (237) | 1110 | - | 72.8% (59) | 27.2% (22) | | 81 |
|  | **EUTIRADS Tot** |  | | | | **4355** |  | | | **6016** |  | | | | **1661** |
|  | | | | | | | | | | |  | | | | |
| ACRTIRADS | ACRTIRADS 1 | <2% | - | 100% (8) | 0% (0) | 8 | - | 100% (19) | 0% (0) | 19 |  | 100% (11) | 0% (0) | | 11 |
|  | ACRTIRADS 2 | <2% | - | 100% (18) | 0% (0) | 18 | - | 99.0% (197) | 1.0% (2) | 199 |  | 98.9% (179) | 1.1% (2) | | 181 |
|  | ACRTIRADS 3 | 5% | >=25 | 92.2% (166) | 7.8% (14) | 180 | - | 94.4% (571) | 5.6% (34) | 605 |  | 95.3% (405) | 4.7% (20) | | 425 |
|  | ACRTIRADS 4 | 5-20 | >=15 | 91.2%(2361) | 8.8 % (227) | 2588 | >=10 | 91.4% (2945) | 8.6% (278) | 3223 |  | 92.0%(584) | 8.0% (51) | | 635 |
|  | ACRTIRADS 5 | >20 | >=10 | 83.1% (1637) | 16.9% (333) | 1970 | >=10 | 83.1% (1637) | 16.9% (333) | 1970 |  |  |  | |  |
|  | **ACRTIRADS Tot** |  | | | | **4764** |  | | | **6016** |  | | | | **1252** |

**Table 5. Sensitivity, Specificity, Positive Predictive Value (PPV), Negative Predictive Value (NPV) for each ultrasound score, and hypothetical percentage of spared FNA depending on each score recommendations. This analysis excluded 458 ATA unclassified nodules for all NUS.**

|  | US Score | Total FNA | Sensitivity | Specificity | PPV (CI95%) | NPV (CI95%) | Spared FNA (%) |
| --- | --- | --- | --- | --- | --- | --- | --- |
| without the ATA unclassified nodules | KTIRADS | 6016 | 94.0% | 11.7% | 11.3%  (+/- 0.8%; 10.5%-12.1%) | 94.1%  (+/- 1.8%; 92.3%-95.9%) | 665 (11.0%) |
|  | ATA | 6016 | 84.5% | 19.5% | 7.7%  (+/- 0.3%; 7.4%-8%) | 94.1%  (+/- 1.7%; 92.4%-95.8%) | 682 (11.3%) |
|  | AACE/ACE-AME | 6016 | 68.4% | 42.2% | 12.5%  (+/- 1.1%; 11.4%-13.6%) | 91.7%  (+/- 0.8%; 90.9%-92.5%) | 2468 (41.0%) |
|  | EUTIRADS | 6016 | 75.7% | 28.0% | 11.2%  (+/- 0.9%; 10.3%-12.1%) | 90.5%  (+/- 1.4%; 89.1%-91.9%) | 1661 (27.6%) |
|  | ACR TIRADS | 6016 | 88.7% | 22.0% | 12.0%  (+/- 0.9%; 11.1%-12.9%) | 94.1%  (+/- 1.3%; 92.8%-95.4%) | 1252 (20.8%) |

**Table 6. Interobserver agreement according to the four US operators.**

|  |  | Operator GP | Operator AC | Operator CP |
| --- | --- | --- | --- | --- |
| K-TIR | **Operator 1*BLINDED*** | 0.59 (+/- 0.08) | 0.57 (+/- 0.08) | 0.59 (+/- 0.07) |
|  | **Operator 2*BLINDED*** |  | 0.61 (+/- 0.08) | 0.55 (+/- 0.09) |
|  | **Operator 3*BLINDED*** |  |  | 0.53 (+/- 0.09) |
| ATA | **Operator 1*BLINDED*** | 0.59 (+/- 0.07) | 0.49 (+/- 0.09) | 0.67 (+/- 0.07) |
|  | **Operator 2*BLINDED*** |  | 0.47 (+/- 0.09) | 0.67 (+/- 0.08) |
|  | **Operator 3*BLINDED*** |  |  | 0.50 (+/- 0.09) |
| AACE | **Operator 1*BLINDED*** | 0.50 (+/- 0.09) | 0.58 (+/- 0.09) | 0.44(+/- 0.09) |
|  | **Operator 2*BLINDED*** |  | 0.52 (+/- 0.09) | 0.57 (+/- 0.08) |
|  | **Operator 3*BLINDED*** |  |  | 0.64 (+/- 0.08) |
| EU-TIR | **Operator 1*BLINDED*** | 0.49 (+/- 0.09) | 0.68 (+/- 0.08) | 0.63 (+/- 0.07) |
|  | **Operator 2*BLINDED*** |  | 0.53 (+/- 0.06) | 0.73 (+/- 0.07) |
|  | **Operator 3*BLINDED*** |  |  | 0.63 (+/- 0.08) |
| ACR-TIR | **Operator 1*BLINDED*** | 0.66 (+/- 0.10) | 0.48 (+/- 0.11) | 0.67 (+/-0.09) |
|  | **Operator 2*BLINDED*** |  | 0.61 (+/- 0.10) | 0.54 (+/- 0.11) |
|  | **Operator 3*BLINDED*** |  |  | 0.62 (+/-0.10) |

**Supplementary material:**

**Figure 1.**

**Panel 1A. ROC curves according to positive cytology from May 2014 for all NUS scores, considering as positive cytology only TIR 4 and TIR5.** Area under ROC curve are: 0.760 (CI95%; 0.720-0.800) for KTIRADS; 0.773 (CI95%; 0.733-0.812) for ATA; 0.764 (CI95%; 0.727-0.801) for AACE/ACE-AME; 0.747 (CI95%; 0.705-0.789) for EUTIRADS; 0.728 (CI95%; 0.688-0.767) for ACR-TIRADS range 1-5

**Panel 1B. ROC curve considering the total scoring of ACRTIRADS (points 1-14).** Area under ROC curve is 0.792 (CI95%; 0.757-0.827).

**
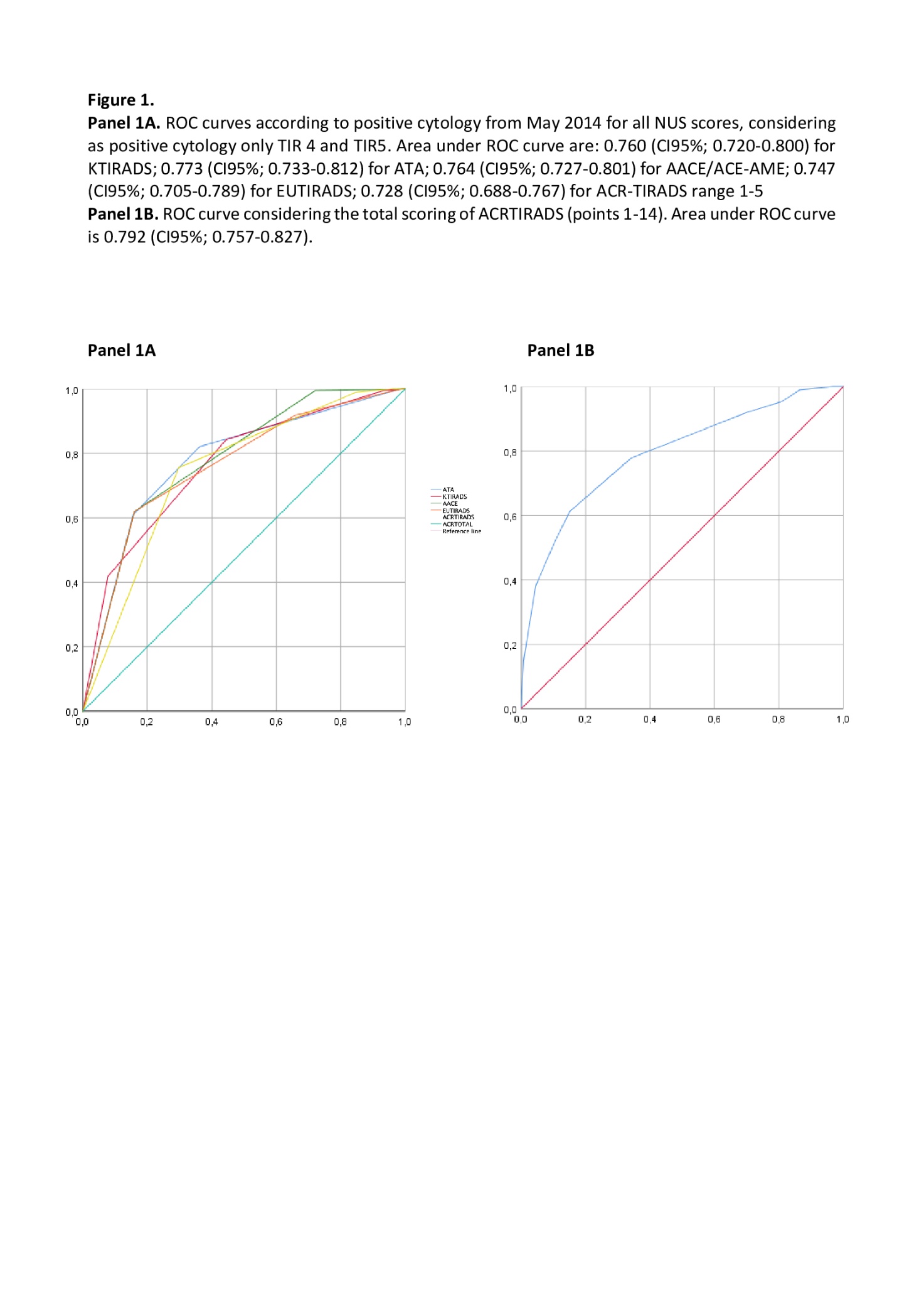
**
